# Canine epidermal lipid sampling by skin scrub revealed variations between different body sites and normal and atopic dogs

**DOI:** 10.1186/1746-6148-10-152

**Published:** 2014-07-10

**Authors:** Mandy Angelbeck-Schulze, Reinhard Mischke, Karl Rohn, Marion Hewicker-Trautwein, Hassan Y Naim, Wolfgang Bäumer

**Affiliations:** 1 Small Animal Clinic, University of Veterinary Medicine Hannover, Foundation, Buenteweg 9, Hanover 30559, Germany; 2 Department of Biometry, Epidemiology and Information Processing, University of Veterinary Medicine Hannover, Foundation, Buenteweg 2, Hanover 30559, Germany; 3 Department of Pathology, University of Veterinary Medicine Hannover, Foundation, Buenteweg 17, Hanover 30559, Germany; 4 Department of Biochemistry, University of Veterinary Medicine Hannover, Foundation, Buenteweg 17, Hanover 30559, Germany; 5 Department of Molecular Biomedical Sciences, NCSU College of Veterinary Medicine, 1060 William Moore Drive, Raleigh, NC 27607, USA

**Keywords:** Canine atopic dermatitis, Epidermal lipids, Ceramides, Skin lipid sampling, Skin scrub, Extraction procedure, Canine, Skin diseases

## Abstract

**Background:**

Previously, we evaluated a minimally invasive epidermal lipid sampling method called skin scrub, which achieved reproducible and comparable results to skin scraping. The present study aimed at investigating regional variations in canine epidermal lipid composition using the skin scrub technique and its suitability for collecting skin lipids in dogs suffering from certain skin diseases. Eight different body sites (5 highly and 3 lowly predisposed for atopic lesions) were sampled by skin scrub in 8 control dogs with normal skin. Additionally, lesional and non-lesional skin was sampled from 12 atopic dogs and 4 dogs with other skin diseases by skin scrub. Lipid fractions were separated by high performance thin layer chromatography and analysed densitometrically.

**Results:**

No significant differences in total lipid content were found among the body sites tested in the control dogs. However, the pinna, lip and caudal back contained significantly lower concentrations of ceramides, whereas the palmar metacarpus and the axillary region contained significantly higher amounts of ceramides and cholesterol than most other body sites. The amount of total lipids and ceramides including all ceramide classes were significantly lower in both lesional and non-lesional skin of atopic dogs compared to normal skin, with the reduction being more pronounced in lesional skin. The sampling by skin scrub was relatively painless and caused only slight erythema at the sampled areas but no oedema. Histological examinations of skin biopsies at 2 skin scrubbed areas revealed a potential lipid extraction from the transition zone between stratum corneum and granulosum.

**Conclusions:**

The present study revealed regional variations in the epidermal lipid and ceramide composition in dogs without skin abnormalities but no connection between lipid composition and predilection sites for canine atopic dermatitis lesions. The skin scrub technique proved to be a practicable sampling method for canine epidermal lipids, revealed satisfying results regarding alterations of skin lipid composition in canine atopic dermatitis and might be suitable for epidermal lipid investigations of further canine skin diseases. Although the ceramide composition should be unaffected by the deeper lipid sampling of skin scrub compared to other sampling methods, further studies are required to determine methodological differences.

## Background

In recent years research on canine epidermal lipids has attracted increasing interest, especially concerning canine atopic dermatitis (AD). Since ceramides (CER) are a major lipid fraction of the stratum corneum (SC) and thought to be responsible for skin barrier homeostasis in mammals [1,2], most studies focused on this group of sphingolipids. Eleven classes of CER have been described in human and canine SC until now [3,4]. Consistently, the different CER classes are denominated according to their chemical structure [5], a sphingoid base - sphingosine (S), dihydrosphingosine (dS), 6-hydroxysphingosine (H) or phytosphingosine (P) -, which is amide linked to a long-chained non- (N), α- (A) or ω-hydroxylated (O) fatty acid. In free ceramides, the ω-hydroxylated fatty acid is further esterified (E), most often with linoleic acid [6]. The 11 classes are CER[EOS], CER[NdS], CER[NS], CER[EOP], CER[NP], CER[EOH], CER[AdS], CER[AS], CER[NH], CER[AP] and CER[AH] (with increasing polarity).

In human and canine AD there is evidence of a defective cutaneous permeability barrier [7-9] which is partially correlated to decreased amounts of CER [4,10-13]. Structural differences in the SC of atopic dogs, which are responsible for the defective skin barrier, do not only exist in affected body areas but also in unaffected skin [14]. Thus, the impaired barrier function seems to be an overall feature of patients suffering from AD. Nevertheless, both human and canine AD are characterised by a classic distribution of lesions [15]. Differences in skin lipid composition at specific body sites may be an explanation for site predispositions of atopic lesions. In humans, regional variations of skin lipid composition and barrier function have been described [16-19], but a correlation between lipid composition and barrier properties was not determined [18]. Furthermore, no connection between lipid composition and body sites predisposed to atopic lesions was detected [19]. In dogs, too, regional variations of permeability barrier properties have been described [20], but until now no studies have been conducted which compared the lipid composition of more than 2 different body regions. Therefore, the investigation of the lipid composition of body sites predisposed for canine AD [21] compared to usually unaffected sites in dogs with healthy skin might reveal interesting facts about the origin of site predispositions.

In a previous study, we demonstrated that a minimally invasive method termed skin scrub is a suitable method for collecting canine epidermal lipids and detected differences in epidermal lipid composition between the inguinal region and the caudal back in dogs with normal skin [22]. The present study used the skin scrub technique to determine regional variations in lipid composition, particularly ceramides, of 8 different body sites in normal dogs. Another important purpose of this study was to test the suitability of skin scrub for the detection of deviations in epidermal lipid composition in certain canine skin diseases, especially atopic dermatitis.

## Methods

### Study design

Samples from 8 different body sites (concave pinna, lip, palmar metacarpus, axillary region, lateral thorax, lateral abdomen, inguinal region, caudal back) were collected, post mortem, by skin scrub from 8 dogs with normal skin. Additionally, skin scrubs were taken from specific lesional and non-lesional areas in 12 atopic dogs and 4 dogs with selected skin disorders other than atopic dermatitis. Lipids were analysed using high performance thin layer chromatography. This study protocol was approved by the Lower Saxony State Office for Consumer Protection and Food Safety (Reference number 09A665).

### Animals

All dogs participating in this study were client-owned. Client consent for study performance was obtained.

The 8 dogs with normal skin were euthanised for reasons not related to this study. They were of different breeds (Labrador Retriever, Border Collie, Dalmatian, Cairn Terrier and 4 mixed breed) with a median age of 12.3 years (4.2–13.3 years). None of the dogs had skin lesions or a history of skin diseases. Dogs that received corticosteroids within 8 weeks or any topical treatment within 1 week prior to sampling were omitted from the study. The integrity of the skin was confirmed by histological examinations of skin biopsies.

The 12 dogs with AD were of different breeds (Jack Russell Terrier, German Shepherd, Golden Retriever, 2 French Bulldogs, American Pit Bull Terrier, Rhodesian Ridgeback, Magyar Vizsla, Dalmatian and 3 mixed breed) with a median age of 3.6 years (1.0–10.5 years). The diagnosis of canine AD was based on appropriate history and clinical criteria according to Favrot et al. [23]. Flea bite hypersensitivity, ectoparasite infestation, hormonal imbalances and secondary bacterial or yeast overgrowth were ruled out by common tests or trial therapies. A food elimination diet had to be performed with each dog for at least 8 weeks. In the end, the 12 atopic dogs included 4 dogs with solely food-induced AD, 4 dogs with non-food-induced AD and 4 dogs with partly food-induced AD.

In addition to the 12 atopic dogs, 4 dogs with other skin diseases participated in this study. A Chow Chow with histologically confirmed sebaceous adenitis, a Poodle-mix with contact dermatitis, and a Small Munsterlander and Landseer with flea bite hypersensitivity. The flea bite hypersensitivity was diagnosed by compatible clinical signs, the finding of fleas or their excrements on the coat and the resolution of clinical signs after ectoparasite treatment.

None of the atopic and non-atopic dogs with skin disorders were treated with anti-inflammatory medications for at least 8 weeks or shampoos for 1 week prior to sampling. One exception was the Landseer with flea bite hypersensitivity, which was treated with a depot methylprednisolone 4 weeks prior to sampling. Thus, sampling was repeated another 4 weeks later.

### Lipid sampling and extraction

Samples from the euthanised dogs with normal skin were taken within 3 hours post mortem from the body sites as described above. Intralesional samples from the atopic dogs included: 9 axillary, 2 inguinal and 1 abdominal. Additionally, from each atopic dog a non-lesional sample was taken from the lateral thoracic region. Intralesional samples from the non-atopic dogs included: the caudal back of the Chow Chow, the ventro-lateral abdomen of the Poodle-mix, and the inguinal region of the Small Munsterlander and the Landseer. Non-lesional samples from the non-atopic dogs were collected from the lateral thorax, with exception of the Chow Chow that was collected from the caudal back. Each body site was sampled once by skin scrub, as described previously [22]. After clipping the coat cautiously, a metal cylinder of 21 mm inner diameter was tightly placed on the skin. The cylinder was filled with 1 millilitre of *n*-hexane and ethanol 2:1 (v/v) which was stirred with a roughened glass rod with slight pressure for 30 s, followed by aspirating the extract into a glass tube. This procedure was performed twice on the same area and the samples were pooled. Immediately after sampling the respective skin areas were washed and a skin lipid complex (Allerderm®, Virbac, Glattbrugg, Switzerland) was applied. The specimens were dried in a SpeedVac (Concentrator Plus, Eppendorf, Hamburg, Germany) at 60°C.

Histological examinations of skin biopsies taken from skin scrubbed areas of 1 euthanised dog showed an approximately 50% reduction in stratum corneum with haematoxylin and eosin stained sections and no lipids in the remaining stratum corneum layers with Sudan III stained sections. Electron microscopic investigations of glutaraldehyde fixed sections showed remaining stratum corneum layers of irregular depth with one partial thin layer remaining with no effect to the stratum granulosum below. Therefore, partial lipid extraction of the transition zone between stratum granulosum and stratum corneum by skin scrub is possible in the areas where only a few stratum corneum layers are present.

Lipid extraction was performed according to Bligh and Dyer [24] by homogenisation of the specimens in methanol, chloroform and distilled water 2:1:0.8 (v/v/v). The addition of 1 millilitre of chloroform and distilled water, respectively, led to phase separation. The upper hydrophilic phase was discarded; the lower lipophilic phase was dried and stored at -20°C until analysis. All solvents used were HPLC grade (Carl Roth, Karlsruhe, Germany).

### High performance thin layer chromatography (HPTLC)

For chromatographical analysis HTPLC-plates (silica gel 60, 20 × 10 cm, Merck, Darmstadt, Germany) were pre-cleaned in chloroform and methanol 1:1 (v/v) and activated in an oven at 110°C for 10 min. The specimens were dissolved in 500 μl of chloroform and methanol 1:1 (v/v). Ten, 5 and 3 microlitres of each specimen were applied by a microlitre syringe (N 701, Hamilton, Bonaduz, Switzerland) in 1 centimetre distance to the bottom of the plate. Additionally, 10 microlitres of each of 6 standard mixtures, which contained increasing concentrations of corresponding lipid standards, were applied to each plate. The following lipid standards were used: phosphatidylcholine, phosphatidylethanolamine, sodium cholesteryl sulphate, galactocerebrosides, α-hydroxy fatty acid ceramide (CER[AS]), non-hydroxy fatty acid ceramide (CER[NS]), oleic acid and glyceryl trioleat (triolein) from Sigma-Aldrich (Steinheim, Germany); Ceramide VI (CER[AP]), Ceramide III (CER[NP]) and Ceramide I (CER[EOS]) from Evonik Industries AG (Essen, Germany); cholesterol and cholesteryl stearate from Fluka Chemie AG (Buchs, Switzerland). Adapting the method described by Stahl et al. [25] for our HPTLC-system, the lipids were separated consecutively in 4 different development chambers (CAMAG, Berlin, Germany) filled with 50 ml of the following solution systems: 1) chloroform, methanol and acetic acid 80:18:2 (v/v/v) to 4 cm, 2) chloroform, methanol and acetic acid 91.4:4.3:4.3 (v/v/v) to 8 cm, 3) *n*-hexane, diethylether and acetic acid 72.7:18.2:9.1 (v/v/v) to 9.5 cm, 4) *n*-hexane to the top. All solvents were HPLC grade and purchased from Carl Roth and Sigma-Aldrich. The plates were dipped into an aquaeous solution of copper sulphate (75 g/l, Merck) and phosphoric acid (8.5%, Sigma-Aldrich) for 5 s, charred in an oven at 170°C for 15 min and scanned (Scanjet G3010, Hewlett-Packard Company, Palo Alto, CA, USA) to allow densitometric analysis using the software programme ImageJ (http://rsb.info.nih.gov/ij). Standard curves obtained by the 6 standard mixtures, applied as described above, were used for the quantification of the respective lipid fractions.

### Statistical analysis

Data distribution was determined by Kolmogorov-Smirnov test and visual assessment of Q-Q plots. All data was calculated with non-parametrical tests, since part of the data did not show standard normal distribution.

Comparisons of the different body sites were done by Friedman test. With significant results pairwise comparisons were performed by Wilcoxon signed-rank test. As the application of an alpha-adjustment did not result in any significant differences, which was inappropriate regarding the results of the global test, its usage was deemed unnecessary.

Wilcoxon two-sample test was used for the comparison of atopic and normal dogs. The epidermal lipids of the lesional area of the atopic dogs (mostly axillary) were compared to the epidermal lipids of the axillary region of the dogs with normal skin. The epidermal lipids of the non-lesional area (thoracic) of the atopic dogs were compared to the epidermal lipids of the lateral thoracic region of the dogs with normal skin. Comparisons of lesional and non-lesional areas of the atopic dogs were done by Wilcoxon signed-rank test.

All analyses were performed using SAS 9.2 (SAS Institute Inc., Cary, NC, USA). Significance level was set at *P* < 0.05.

## Results

### Comparison of canine epidermal lipids at different body sites in normal skin

No statistically significant differences were detected in total lipid content between the 8 tested body sites (see Additional file 1). In contrast, significant differences in the absolute amounts of CER were found among 6 of the 8 body sites tested (*P* < 0.01, Friedman test) with higher amounts at the metacarpus, axillary region and lateral thorax and lower amounts at the pinna, lip and caudal back (see Additional file 1). The ceramide classes also differed significantly in content between the body sites except for CER[NP] (see Additional file 1). Significant differences in ceramide classes occurred between canine AD predilection sites (pinna and metacarpus) as well as between non-predilection sites (lateral thorax and caudal back). No significant differences in ceramide classes were found among the palmar metacarpus, axillary region and lateral thorax or between the axillary and inguinal region (see Additional file 1). Most differences were found for CER[AS+NH] (*P* < 0.001) with significantly lower levels at the pinna, lip and caudal back compared to most other sites. The levels of CER[EOS] were significantly lower at the pinna and lip when compared to the metacarpus, axillary and inguinal region. The levels of CER[EOP] were significantly lower at the caudal back than at other body sites (see Additional file 1).

Among the remaining lipid fractions, no significantly different concentrations of glucosylceramides and free fatty acids were found among the body sites. The absolute values of phospholipids, phosphatidylethanolamine, cholesterol sulphate, cholesterol and cholesteryl esters differed significantly among the tested body sites (*P* < 0.01, Friedman test) as well as did the amounts of triglycerides (*P* < 0.0001, Friedman test; see Additional file 1). The pinna contained significantly lower amounts of cholesterol and cholesteryl esters but significantly higher amounts of triglycerides compared to other body sites. The skin at the palmar metacarpus and axillary region contained significantly higher amounts of cholesterol compared to other body sites. The concentrations of cholesterol sulphate and triglycerides were significantly lower in abdominal and inguinal skin compared to other body sites. The skin at the caudal back contained significantly higher concentrations of cholesteryl esters compared to other body sites (see Additional file 1). Similar to the ceramide composition, no specific epidermal lipid composition was detected in body sites with high predisposition for atopic lesions compared to sites with low predisposition for atopic lesions.

The ratio of ceramides to cholesterol was significantly lower at the caudal back compared to the inguinal region and pinna (data not shown).

### Differences in canine epidermal lipids between lesional and non-lesional skin of atopic dogs compared to normal skin

**Figure 1:**
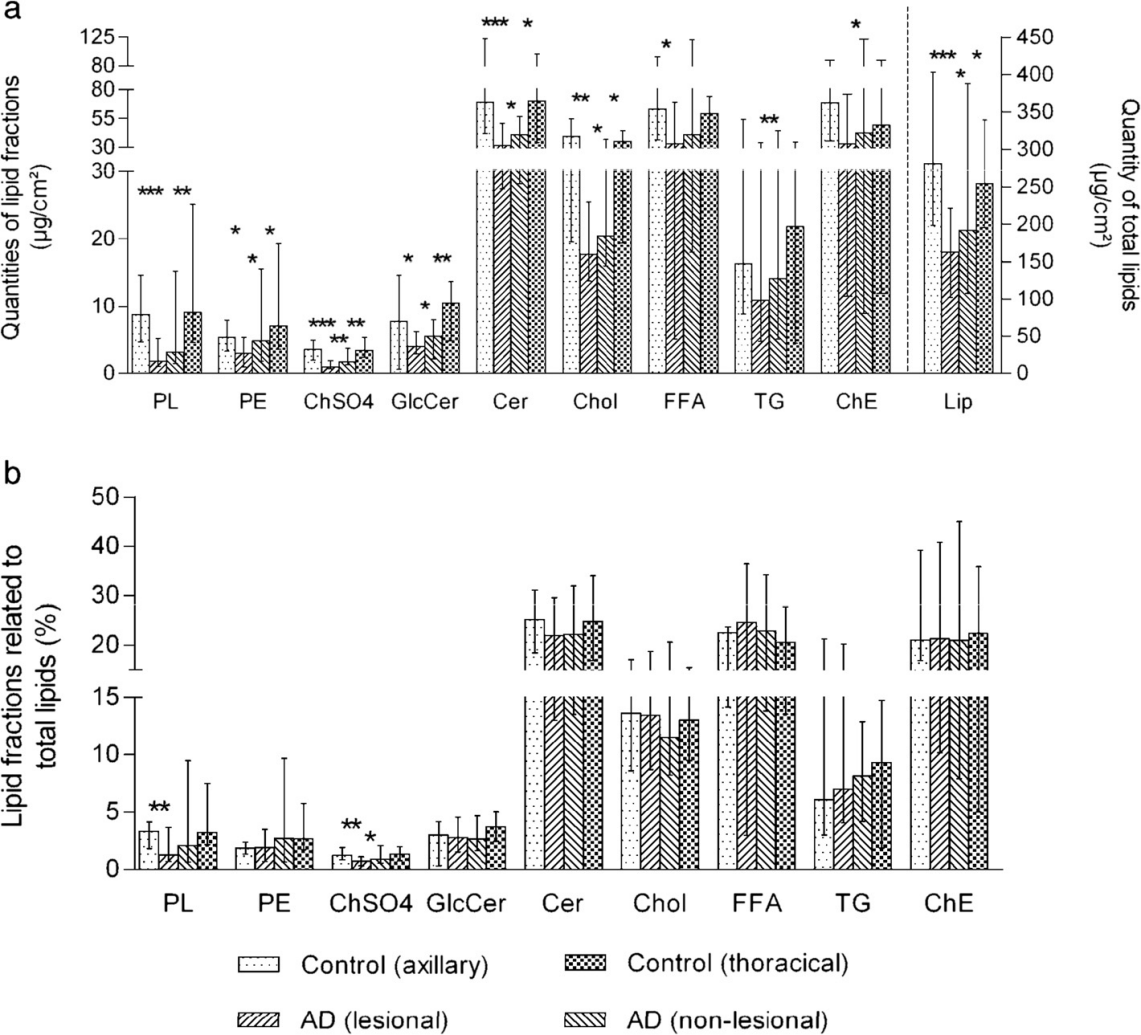
**Comparison of absolute (a) and relative (b) amounts of lipid fractions between atopic and control dogs.** PL, phospholipids; PE, phosphatidylethanolamine; ChSO4, cholesterol sulphate; GlcCer, glucosylceramides; Cer, ceramides; Chol, cholesterol; FFA, free fatty acids; TG, triglycerides; ChE, cholesteryl ester; Lip, total lipids; *P < 0.05; **P < 0.01; ***P < 0.001.

**Figure 2:**
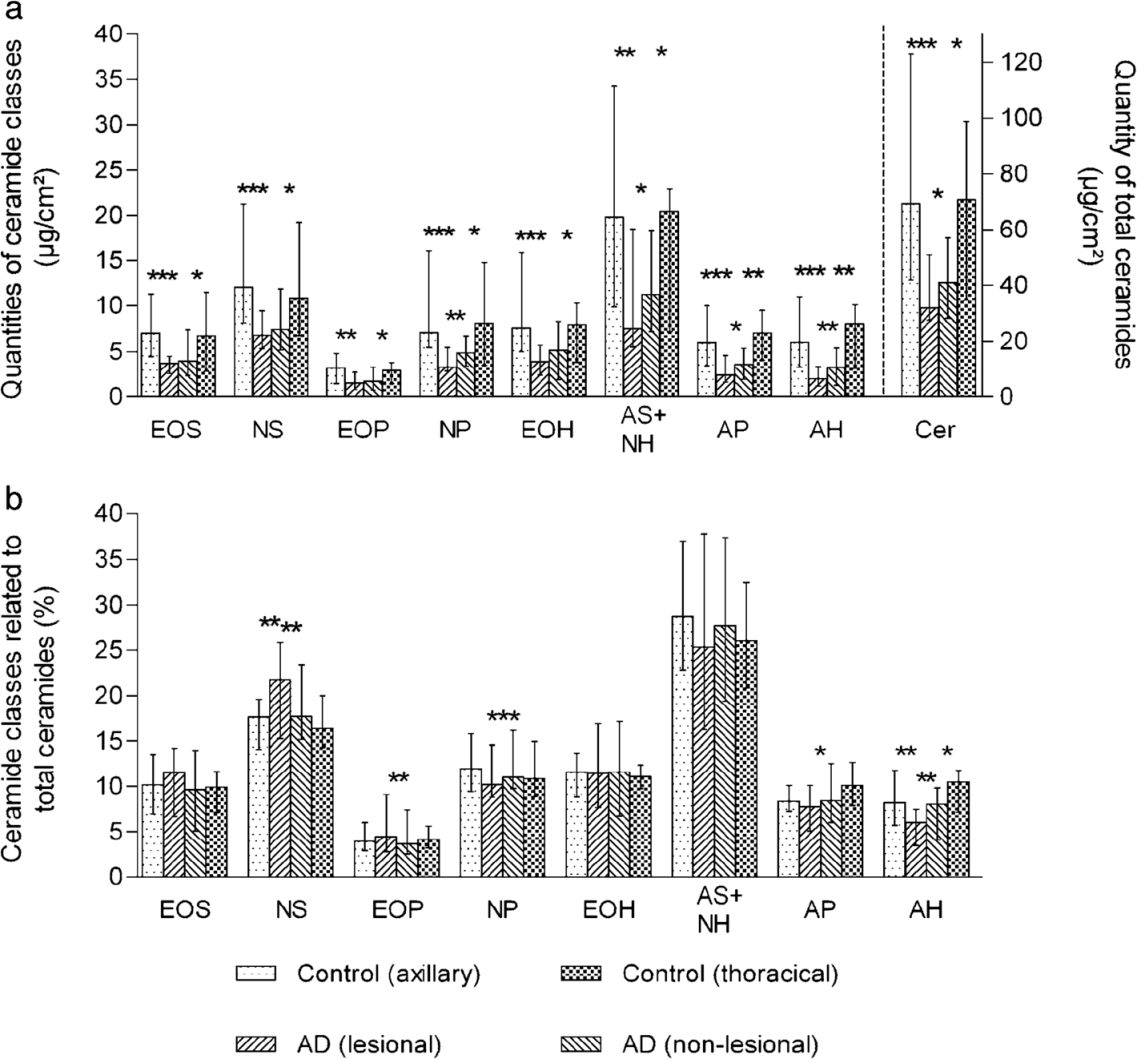
**Comparison of absolute (a) and relative (b) amounts of ceramide classes between atopic and control dogs.** EOS, ceramide from sphingosine and esterified ω-hydroxy fatty acid (FA); NS, ceramide from sphingosine and non-hydroxy FA; EOP, ceramide from phytosphingosine and esterified ω-hydroxy FA; NP, ceramide from phytosphingosine and non-hydroxy FA; EOH, ceramide from 6-hydroxy sphingosine and esterified ω-hydroxy FA; AS+NH, ceramide from sphingosine and α-hydroxy FA and ceramide from 6-hydroxy sphingosine and non-hydroxy FA; AP, ceramide from phytosphingosine and α-hydroxy FA; AH, ceramide from 6-hydroxy sphingosine and α-hydroxy FA; Cer, total ceramides; *P < 0.05; **P < 0.01; ***P < 0.001.

The total amount of lipid was significantly lower in both lesional and non-lesional areas in atopic dogs compared to normal canine skin (Figure 1a). In a similar manner, the contents of lipid fractions (except for free fatty acids, triglycerides and cholesteryl esters) were significantly reduced in atopic dogs compared to normal canine skin. Samples of the lesional regions contained significantly lower amounts of all lipid fractions compared to the samples of the non-lesional regions, except for phospholipids and free fatty acids (Figure 1a). By contrast, rare significant differences in the percentage lipid composition between atopic and control dogs were determined (Figure 1b). No differences were detected between the ratios of ceramides to cholesterol in atopic and normal skin (data not shown).The skin of atopic dogs contained significantly lower amounts of all ceramide classes than normal canine skin (Figure 2a). Additionally, the contents of CER[AH], CER[AP], CER[AS+NH] and CER[NP] were significantly reduced in lesional skin compared to non-lesional skin. The differences between lesional and normal skin were more pronounced than between non-lesional and normal skin (Figure 2a). By contrast, rare significant differences in the ceramide profile between atopic and normal dogs were detected (Figure 2b).

No remarkable differences were noted in lipid or ceramide composition between the three subgroups of canine AD (solely food-induced, partly food-induced, non-food-induced; data not shown).

### Lipid composition of other skin disorders

Total lipid content and the amount of all lipid fractions and ceramide classes were markedly reduced in the lesional skin of the dog with contact dermatitis compared to its non-lesional skin and normal skin, whereas only the total ceramides and all ceramide classes were reduced in non-lesional skin compared to normal skin (see Additional file 2).

In the 2 dogs with flea bite hypersensitivity, the concentrations of total ceramides, all ceramide classes and cholesterol were reduced in both lesional and non-lesional skin compared to normal skin with no differences detected between lesional and non-lesional skin (see Additional file 2). Additionally, there were decreased levels of total ceramides, all ceramide classes and cholesterol in both lesional and non-lesional skin of the Landseer with flea bite hypersensitivity 8 weeks after the systemic administration of a time-released corticosteroid compared to 4 weeks after the administration. At the latter time of sampling the quantities of all lipid fractions were comparable to those of normal skin (data not shown).

The lesional skin of the dog with sebaceous adenitis contained lower amounts of total lipids, free fatty acids, triglycerides and cholesteryl esters compared to its non-lesional skin or normal skin. Interestingly, the amount of cholesteryl esters in the lesional skin was one tenth of that in the non-lesional skin. Furthermore, there was no difference in total ceramides and ceramide classes when compared to normal skin (see Additional file 2).

### Toleration of the skin scrub technique

Sampling was feasible without sedation and with relatively low restraint of the dogs as excessive struggling did not occur. The skin of the dogs tested in our study appeared erythematous with no visible oedema at the sites sampled. Six dogs scratched at the sampled areas for 1 to 2 days. In 9 cases the sampled area of non-lesional skin developed a crusty circle that resolved 2 to 4 days later without treatment.

## Discussion

Canine AD is characterised by a classic distribution of lesions [15]. Commonly affected body sites in canine AD are the face, pinnae, paws, axillary, inguinal and flexural regions [21,23]. To determine a possible predisposition of such sites for the development of lesions in canine AD we investigated the epidermal lipids in dogs with normal skin at 8 different body sites, 5 of which are typically affected in canine AD (concave pinna, lip, palmar metacarpus, axillary and inguinal region) and 3 of which are usually not affected (lateral thorax and abdomen, caudal back). However, there were neither any differences detectable in the total lipid content between the body sites nor a clear connection between the groups of body sites mentioned above and the quantities of particular lipid fractions, especially ceramides or ceramide classes. These findings correspond with the results of a similar study concerning human epidermal lipid composition [19]. We concluded that the site predispositions for atopic lesions in dogs appear unrelated to a lower lipid or ceramide content but may be due to other factors that lead to a more intense allergen contact such as lesser hair coat and higher mechanical stress of these specific regions.

To the best of the authors’ knowledge this is the first study that compared the quantities of lipid and ceramide fractions at more than 2 different body sites in normal canine skin. Although no differences in lipid content were present, the lipid composition was not uniform across the body sites. The variations in triglyceride and cholesteryl ester content are explicable by regional variations of sebaceous glands [26], because these lipids are mainly derived from sebum [27]. This was also confirmed by the lower amounts of triglycerides and cholesteryl esters in the lesional skin in the dog with sebaceous adenitis compared to its non-lesional skin.

The high concentrations of cholesterol sulphate, cholesterol and sphingolipids at the palmar metacarpus compared to other body sites determined in this study correlate to the regional variations in epidermal lipid composition found in humans at the sole of the foot compared to other body sites [28]. Furthermore, there is evidence that the lipid composition is prone to changes during the seasons, which further modifies the regional variations [19]. This aspect has not been investigated in dogs and was not taken into account in this study. The wide inter-individual variation of lipid content and composition, which is shown in Table S1 (see Additional file 1), has been described similarly in the epidermal structure of dogs [29]. Unfortunately, wide variations between different individuals are able to obliterate the effect of site dependences [18].

Although there was no connection between the predisposition of body sites for atopic lesions and their lipid composition in normal canine skin, the lipid content and composition differed significantly between lesional and non-lesional skin in atopic dogs. Total lipids and especially ceramide classes were significantly lower in concentration in lesional than in non-lesional skin of the atopic dogs tested in the present study. However, this lipid reduction might be due to inflammation processes in lesional skin. There is evidence that allergen exposition leading to inflammation in the affected skin areas worsens the epidermal barrier properties of experimentally sensitized dogs [20,30]. Additionally, Hightower et al. [20] detected a higher transepidermal water loss (TEWL) at 7 out of 10 body sites with 6 of these sites being predisposed to AD lesions in the sensitized dogs compared to normal dogs prior to the allergen challenge (i.e. prior to the development of skin lesions in the sensitized dogs). Since Shimada et al. [13] indicated a negative correlation between TEWL and ceramide content in the skin of atopic dogs, one can presume lower ceramide contents in canine AD predilection sites prior to an allergen contact. This altered lipid composition may trigger a more rapid inflammation leading to atopic lesions in contrast to body sites that are not predisposed to AD lesions.

In contrast to former studies dealing with canine epidermal lipids [4,13,30], the present study revealed significant differences in lipid composition between lesional and non-lesional skin in atopic dogs. In the former studies, non-lesional skin was sampled quite close to the area of the sampled lesional skin region [4,13,30]. This may explain the lack of differences between lesional and non-lesional skin in atopic dogs in these studies. Therefore, we chose to sample the lateral thorax for a non-lesional area and mostly the axillary region as a lesional area in atopic dogs. Since we found no substantial differences between these body sites in normal dogs, the comparison between lesional and non-lesional skin in atopic dogs was made irrespective of the body site.

The ability to compare the lipid composition at different body sites of the same individuals, such as lesional and non-lesional skin, is an advantage of minimally invasive sampling methods, like the skin scrub technique. This technique was originally performed with a detergent instead of a solvent and successfully applied to canine skin to determine the cutaneous bacterial flora [31]. In that study, tractable dogs were sampled without anaesthesia by using minimal restraint, as in accordance with our experience during the skin lipid sampling of dogs in this study. Furthermore, mixtures of hexane and alcohols have been used to extract human and porcine epidermal lipids with minimal irritation [18,32-34], which corresponds with our observations. Thus, the skin scrub technique seems to be a practicable, relatively painless method for skin lipid sampling in dogs.

Despite this fact, we decided to sample recently deceased dogs as a control and to compare 8 different body sites, some of which would have been difficult to sample in living dogs (i.e. concave pinna, lip and palmar metacarpus). A critical argument concerning our study results may be the comparison of deceased and living dogs owing to a possibly less intense scrubbing in atopic (i.e. living) dogs than in normal (i.e. deceased) dogs during skin sampling. To control for this possible artifact, sampling was always performed by the same person, who took care to scrub with the same pressure in each case. Another influencing factor that may distort our results was the distinctly higher median age of the control dogs compared to the atopic dogs. There is only one study concerning the epidermal lipid composition in dogs correlated to age, in which it examined the skin surface lipids in young dogs at the age of 10 to 81 weeks [35]. To the best of the authors’ knowledge, no literature is available concerning the skin lipid composition in older dogs. Owing to the fact that the lipid and ceramide contents are decreased in the aged skin of humans [36], one may expect the same for canine aged skin. Thus, the amounts of total lipids and ceramides may be lower in our control dogs compared to dogs that would match the age of the atopic dogs tested in the present study. In this case, the differences between atopic and normal dogs would have been even more pronounced.

Contrary to our results, there are two studies that found significant changes regarding relative ceramide content or composition in atopic dogs [12,13]. A possible explanation for these discrepancies may be the different ways used to determine the ceramide content. In the present study, percentage values of the lipid fractions were calculated based on the sum of all lipid fractions that we measured (lipid fractions shown in Figure 1). Whereas Shimada et al. [13] determined the sum of 2 ceramide classes as ceramide content and calculated the percentage values based on the sum of ceramides, cholesterol and free fatty acids. The percentage values for the ceramide classes in the present study were exclusively based on the sum of 9 ceramide classes, while Reiter et al. [12] used percentage values based on 5 ceramide classes and cholesterol. The different sampling, analytical and quantification methods may also contribute to the differences in the study results. Nevertheless, Shimada et al. [13] showed a strikingly decreased ceramide content in their chromatograms of lesional and non-lesional skin in dogs with atopic dermatitis compared to normal canine skin, while the cholesterol content was similar. We only found slight differences in ceramide:cholesterol ratios between normal and atopic canine skin in our study (1.90 and 2.03 for axillary and thoracic region of normal canine skin, respectively; 1.72 and 1.85 for lesional and non-lesional atopic canine skin, respectively). The reason for this deviation in our results compared to the former study remains unclear.

In contrast to the relative comparisons, the present study revealed a marked decrease in total lipids, more precisely of all ceramide classes and most lipid fractions, in both lesional and non-lesional skin of atopic dogs compared to normal dogs. These results are mainly in accordance with a more recent study [4] which exclusively investigated the ceramide composition of canine atopic skin compared to normal controls and found significantly lower absolute amounts of total ceramides and the ceramide classes CER[EOS], CER[NS+NdS], CER[EOP], CER[NP] and CER[AS+NH] in both lesional and non-lesional skin of atopic dogs. However, there are slight discrepancies between their results and ours that may be due to varying study protocols including the sampling method, different ways of quantification (μg/mg versus μg/cm^2^ stratum corneum) and different statistical methods. The comparison of our results with former studies is further complicated by the sampling method; since tape stripping used by Popa et al. [11] and Yoon et al. [4], cyanoacrylate stripping used by Reiter et al. [12] and Stahl et al. [30] as well as solvent extraction used by Shimada et al. [13] only harvest stratum corneum lipids. As our histological examinations revealed, the skin scrub technique probably also extracts lipids from the transition zone between stratum granulosum and stratum corneum. This may explain the presence of glucosylceramides in normal canine skin in our study. Popa et al. [11] detected significant amounts of glucosylceramides exclusively in atopic canine skin with concurrently decreased amounts of ceramides and suggested an impaired β-glucocerebrosidase-activity as an explanation. However, tape stripping used by Popa et al. [11] may not reach the transition from stratum granulosum to stratum corneum, which explains the lack of glucosylceramides in normal dog skin. The significant amounts of glucosylceramides in atopic canine skin described by Popa et al. [11] may originate from the delayed and disordered release of lipids from lamellar bodies to the intercellular space of the stratum corneum in atopic canine skin [15]. If the glucosylceramides accumulated in the stratum corneum in dogs with atopic dermatitis while ceramides decreased, the glucosylceramide:ceramide ratio would increase. This was not observed in our study (0.12 and 0.14 for axillary and thoracic region of normal canine skin, respectively; 0.14 and 0.13 for lesional and non-lesional atopic canine skin, respectively). Consequently, it has to be considered that the total amount of glucosylceramides of normal and atopic dog skin is unaltered and that only the distribution of glucosylceramides is different in atopic canine skin compared to normal dog skin. The decreased measurements of glucosylceramides in atopic canine skin compared to normal canine skin in our study might result from retaining lamellar bodies in corneocytes [15]. The results of our study would be rather compatible with an increased activity of sphingomyelin-glucosylceramide-deacylase as the underlying mechanism for the ceramide deficiency in canine atopic skin as described in human atopic dermatitis [37]. Furthermore, our results are consistent with human skin lipid studies that also detected decreased absolute amounts of total lipids and total ceramides in atopic patients [36] but no differences in relative lipid composition [36,38].

However, the findings regarding the decreased lipid and/or ceramide contents do not seem to be unique for AD. Similar alterations of the ceramide composition were also detected in other human skin diseases, such as psoriasis [1]. In the present study, there is evidence for analogical observations of other canine skin diseases, as we detected similarly decreased amounts of the ceramides including all subclasses in the lesional and non-lesional skin in the Poodle-mix with contact dermatitis as well as in the 2 dogs with flea bite hypersensitivity compared to normal skin. Although the validity of these results is only related to individual cases, such alterations of the epidermal lipid composition in other canine skin diseases may be indicative of changes secondary to inflammation (allergic or immunological mediated). This hypothesis is further confirmed by the observation of Stahl et al. [30], that the decreased ceramide levels after allergen challenge returned back to prechallenge values within 2 months after lesion resolution in house dust mite sensitized atopic Maltese-Beagle dogs. These observations provide interesting aspects for further studies, as indicated by Olivry et al. [9].

Regarding the clinical perspective of this study, one may suspect a beneficial effect of a topically applied lipid preparation on atopic canine skin. A few studies [39-41] gave evidence for the benefit of (topical or systemic) application of lipid preparations to atopic dogs. With regard to our investigations, we would assume the most beneficial effect on canine atopic skin by using a topically applied lipid formulation which contains skin lipids in the same amounts as they exist in normal canine skin. Interestingly, such lipid preparations might also be beneficial for dogs with skin diseases like contact or flea bite dermatitis. For a more precise statement, further investigations would be necessary.

## Conclusions

In conclusion, we found regional variations in lipid composition in the canine epidermis but no connection between the lipid or ceramide composition and canine AD predilection sites. In comparison to body site-specific controls, we detected marked alterations of lipid contents and composition, especially of ceramides, in lesional and non-lesional skin of atopic dogs. The question whether the lipid alterations and the resulting impaired barrier function in canine atopic skin are of primary or secondary origin remains unanswered. Finally, the skin scrub technique proved to be a practicable sampling method for canine epidermal lipids and revealed reproducible results regarding alterations of skin lipid composition in canine atopic dermatitis. This sampling method may also be suitable for epidermal lipid investigations in further canine skin diseases. Due to its laboratory advantages (large sample quantity, simple sample preparation), the skin scrub technique may be used as an alternative canine skin lipid sampling method in the future, even though lipids from the transition zone between stratum corneum and stratum granulosum may be co-extracted. Since the intercellular stratum corneum lipids are generated from the more polar lipids at the transition from stratum granulosum to stratum corneum [2], the investigation of these more polar lipids might also be important for further understanding of barrier impairment in canine atopic dermatitis. However, for a precise evaluation of the skin scrub technique, comparative studies of this sampling method with tape stripping and cyanoacrylate stripping need to be conducted.

## Abbreviations

A: α-hydroxy fatty acid; AD: Atopic dermatitis; CER: Ceramides; CER[XY]: Ceramide class consisting of sphingoid base Y amide linked to fatty acid X; dS: Dihydrosphingosine; E: Esterified fatty acid; H: 6-hydroxy sphingosine; HPLC: High pressure liquid chromatography; HPTLC: High performance thin layer chromatography; N: Non-hydroxy fatty acid; O: ω-hydroxy fatty acid; P: Phytosphingosine; S: Sphingosine; SC: Stratum corneum; TEWL: Transepidermal water loss.

## Competing interests

The authors declare that they have no competing interests.

## Authors’ contributions

MAS conceived the study, performed the skin lipid sampling and the measurements, analysed the data and prepared the manuscript. KR performed the statistical analysis. HYN participated in the study design and supervised the measurements. MHT conducted the histological examinations. RM and WB conceived the study, participated in the study design and coordination and revised the manuscript. All authors read and approved of the final manuscript.

## Supplementary Material

Additional file 1: Table S1 Comparison of epidermal lipid composition between eight different body sites sampled by skin scrub. Click here for file

Additional file 2: Table S2 Epidermal lipid composition of individual dogs with selected skin diseases in comparison with body site matched controls. Click here for file

## References

[B1] Coderch L Lopez O de la Maza A Parra JL Ceramides and skin function Am J Clin Dermatol 2003 4 107 129 10.2165/00128071-200304020-00004 12553851

[B2] Holleran WM Takagi Y Uchida Y Epidermal sphingolipids: metabolism, function, and roles in skin disorders FEBS letters 2006 580 5456 5466 10.1016/j.febslet.2006.08.039 16962101

[B3] Masukawa Y Narita H Shimizu E Kondo N Sugai Y Oba T Homma R Ishikawa J Takagi Y Kitahara T Takema Y Kita K Characterization of overall ceramide species in human stratum corneum J Lip Res 2008 49 1466 1476 10.1194/jlr.M800014-JLR200 18359959

[B4] Yoon JS Nishifuji K Sasaki A Ide K Ishikawa J Yoshihara T Iwasaki T Alteration of stratum corneum ceramide profiles in spontaneous canine model of atopic dermatitis Exp Dermatol 2011 20 732 736 10.1111/j.1600-0625.2011.01306.x 21649737

[B5] Motta S Monti M Sesana S Caputo R Carelli S Ghidoni R Ceramide composition of the psoriatic scale Biochim Biophys Acta 1993 1182 147 151 10.1016/0925-4439(93)90135-N 8357845

[B6] Novotny J Hrabalek A Vavrova K Synthesis and structure-activity relationships of skin ceramides Curr Med Chem 2010 17 2301 2324 10.2174/092986710791331068 20459376

[B7] Choi MJ Maibach HI Role of ceramides in barrier function of healthy and diseased skin Am J Clin Dermatol 2005 6 215 223 10.2165/00128071-200506040-00002 16060709

[B8] Nishifuji K Yoon JS The stratum corneum: the rampart of the mammalian body Vet Dermatol 2013 24 60 e16 10.1111/j.1365-3164.2012.01090.x 23331681

[B9] Olivry T Is the skin barrier abnormal in dogs with atopic dermatitis? Vet Immunol Immunopathol 2011 144 11 16 10.1016/j.vetimm.2011.07.014 21835476

[B10] Imokawa G Lipid abnormalities in atopic dermatitis J Am Acad Dermatol 2001 45 Suppl 1 29 32 10.1067/mjd.2001.117020 11423869

[B11] Popa I Remoue N Hoang L Pin D Gatto H Haftek M Portoukalian J Atopic dermatitis in dogs is associated with a high heterogeneity in the distribution of protein-bound lipids within the stratum corneum Arch Dermatol Res 2011 303 433 440 10.1007/s00403-011-1120-5 21240511

[B12] Reiter LV Torres SM Wertz PW Characterization and quantification of ceramides in the nonlesional skin of canine patients with atopic dermatitis compared with controls Vet Dermatol 2009 20 260 266 10.1111/j.1365-3164.2009.00759.x 19659537

[B13] Shimada K Yoon JS Yoshihara T Iwasaki T Nishifuji K Increased transepidermal water loss and decreased ceramide content in lesional and non-lesional skin of dogs with atopic dermatitis Vet Dermatol 2009 20 541 546 10.1111/j.1365-3164.2009.00847.x 20178492

[B14] Marsella R Olivry T Carlotti DN Current evidence of skin barrier dysfunction in human and canine atopic dermatitis Vet Dermatol 2011 22 239 248 10.1111/j.1365-3164.2011.00967.x 21414049

[B15] Marsella R Samuelson D Unravelling the skin barrier: a new paradigm for atopic dermatitis and house dust mites Vet Dermatol 2009 20 533 540 10.1111/j.1365-3164.2009.00809.x 20178491

[B16] Kleesz P Darlenski R Fluhr JW Full-body skin mapping for six biophysical parameters: baseline values at 16 anatomical sites in 125 human subjects Skin Pharmacol Physiol 2012 25 25 33 10.1159/000330721 21912200

[B17] Lampe MA Williams ML Elias PM Human epidermal lipids: characterization and modulations during differentiation J Lip Res 1983 24 131 140 6833890

[B18] Norlen L Nicander I Lundh Rozell B Ollmar S Forslind B Inter- and intra-individual differences in human stratum corneum lipid content related to physical parameters of skin barrier function in vivo J Invest Dermatol 1999 112 72 77 10.1046/j.1523-1747.1999.00481.x 9886267

[B19] Yoshikawa N Imokawa G Akimoto K Jin K Higaki Y Kawashima M Regional analysis of ceramides within the stratum corneum in relation to seasonal changes Dermatology 1994 188 207 214 10.1159/000247141 8186510

[B20] Hightower K Marsella R Flynn-Lurie A Effects of age and allergen exposure on transepidermal water loss in a house dust mite-sensitized beagle model of atopic dermatitis Vet Dermatol 2010 21 89 96 10.1111/j.1365-3164.2009.00839.x 20187914

[B21] Jaeger K Linek M Power HT Bettenay SV Zabel S Rosychuk RA Mueller RS Breed and site predispositions of dogs with atopic dermatitis: a comparison of five locations in three continents Vet Dermatol 2010 21 118 122 20187918 10.1111/j.1365-3164.2009.00845.x

[B22] Angelbeck-Schulze M Stahl J Brodesser S Rohn K Naim H Hewicker-Trautwein M Kietzmann M Bäumer W Mischke R Comparison of three different sampling methods for canine skin lipids Vet Dermatol 2013 24 e233 e251 10.1111/vde.12015 23470179

[B23] Favrot C Steffan J Seewald W Picco F A prospective study on the clinical features of chronic canine atopic dermatitis and its diagnosis Vet Dermatol 2010 21 23 31 10.1111/j.1365-3164.2009.00758.x 20187911

[B24] Bligh EG Dyer WJ A rapid method of total lipid extraction and purification Can J Biochem Physiol 1959 37 911 917 10.1139/o59-099 13671378

[B25] Stahl J Niedorf F Kietzmann M Characterisation of epidermal lipid composition and skin morphology of animal skin ex vivo Eur J Pharm Biopharm 2009 72 310 316 10.1016/j.ejpb.2008.09.013 18940254

[B26] Pavletic MM Slatter DH The Integument Textbook of Small Animal Surgery 2003 2 3 Philadelphia: Saunders 250 257

[B27] Yoon JS Nishifuji K Ishioroshi S Ide K Iwasaki T Skin lipid profiling in normal and seborrhoeic shih tzu dogs Vet Dermatol 2013 24 84 e22 10.1111/j.1365-3164.2012.01102.x 23331684

[B28] Lampe MA Burlingame AL Whitney J Williams ML Brown BE Roitman E Elias PM Human stratum corneum lipids: characterization and regional variations J Lip Res 1983 24 120 130 6833889

[B29] Lloyd DH Garthwaite G Epidermal structure and surface topography of canine skin Res Vet Sci 1982 33 99 104 7134655

[B30] Stahl J Paps J Bäumer W Olivry T Dermatophagoides farinae house dust mite allergen challenges reduce stratum corneum ceramides in an experimental dog model of acute atopic dermatitis Vet Dermatol 2012 23 497 e497 10.1111/j.1365-3164.2012.01114.x 23140315

[B31] Ihrke PJ Schwartzman RM McGinley K Horwitz LN Marples RR Microbiology of normal and seborrheic canine skin Am J Vet Res 1978 39 1487 1489 151523

[B32] Bonte F Pinguet P Chevalier JM Meybeck A Analysis of all stratum corneum lipids by automated multiple development high-performance thin-layer chromatography J Chromatogr B Biomed Appl 1995 664 311 316 10.1016/0378-4347(94)00480-S 7780582

[B33] Farwanah H Raith K Neubert RH Wohlrab J Ceramide profiles of the uninvolved skin in atopic dermatitis and psoriasis are comparable to those of healthy skin Arch Dermatol Res 2005 296 514 521 10.1007/s00403-005-0551-2 15803327

[B34] Monteiro-Riviere NA Inman AO Mak V Wertz P Riviere JE Effect of selective lipid extraction from different body regions on epidermal barrier function Pharm Res 2001 18 992 998 10.1023/A:1010944529387 11496960

[B35] Dunstan RW Herdt TH Olivier B Mei BS Credille KM Kennis RA Maier RL Olivier B Castle S Reinhart GA Davenport GM Thoday KL, Foil CS, Bond R Age- and breed-related differences in canine skin surface lipids and pH Advances in veterinary dermatology Vol 4, Proceedings of the Fourth World Congress of Veterinary Dermatology: 30 August - 2 September 2000; San Francisco, California, USA 2002 Oxford: Blackwell 37 42

[B36] Imokawa G Abe A Jin K Higaki Y Kawashima M Hidano A Decreased level of ceramides in stratum corneum of atopic dermatitis: an etiologic factor in atopic dry skin? J Invest Dermatol 1991 96 523 526 10.1111/1523-1747.ep12470233 2007790

[B37] Imokawa G A possible mechanism underlying the ceramide deficiency in atopic dermatitis: expression of a deacylase enzyme that cleaves the N-acyl linkage of sphingomyelin and glucosylceramide J Dermatol Sci 2009 55 1 9 10.1016/j.jdermsci.2009.04.006 19443184

[B38] Yamamoto A Serizawa S Ito M Sato Y Stratum corneum lipid abnormalities in atopic dermatitis Arch Dermatol Res 1991 283 219 223 10.1007/BF01106105 1929538

[B39] Piekutowska A Pin D Reme CA Gatto H Haftek M Effects of a topically applied preparation of epidermal lipids on the stratum corneum barrier of atopic dogs J Comp Path 2008 138 197 203 10.1016/j.jcpa.2008.01.006 18374938

[B40] Popa I Pin D Remoué N Osta B Callejon S Videmont E Gatto H Portoukalian J Haftek M Analysis of epidermal lipids in normal and atopic dogs, before and after administration of an oral omega-6/omega-3 fatty acid feed supplement. A pilot study Vet Res Commun 2011 35 501 509 10.1007/s11259-011-9493-7 21786009

[B41] Popa I Remoue N Osta B Pin D Gatto H Haftek M Portoukalian J The lipid alterations in the stratum corneum of dogs with atopic dermatitis are alleviated by topical application of a sphingolipid-containing emulsion Clin Exp Dermatol 2012 37 665 671 10.1111/j.1365-2230.2011.04313.x 22360796

