# Supplementary material for: Canine epidermal lipid sampling by skin scrub revealed variations between different body sites and normal and atopic dogs
Source: BMC Vet Res. 2014 Jul 10;10:152. doi: 10.1186/1746-6148-10-152 (PMC4107596; doi:10.1186/1746-6148-10-152)
Supplement: Additional file 1: Table S1 — Comparison of epidermal lipid composition between eight different body sites sampled by skin scrub. [file 1746-6148-10-152-S1.doc]

Table 1: Comparison of epidermal lipid composition between eight different body sites sampled by skin scrub.

All values are given in µg/cm² epidermis (median, minimum – maximum). PL, phospholipids; PE, phosphatidylethanolamin; ChSO4, cholesterol sulphate; GlcCer, glucosylceramides; CER, ceramides; [EOS], ceramide from sphingosine and esterified ω-hydroxy fatty acid (FA); [NS], ceramide from sphingosine and non-hydroxy FA; [EOP], ceramide from phytosphingosine and esterified ω-hydroxy FA; [NP], ceramide from phytosphingosine and non-hydroxy FA; [EOH], ceramide from 6-hydroxy sphingosine and esterified ω-hydroxy FA; [AS+NH] ceramide from sphingosine and α-hydroxy FA and ceramide from 6-hydroxy sphingosine and non-hydroxy FA; [AP], ceramide from phytosphingosine and α-hydroxy FA; [AH], ceramide from 6-hydroxy sphingosine and α-hydroxy FA; Chol, cholesterol; FFA, free fatty acids; TG, triglycerides; ChE, cholesteryl ester; ns, non-significant; * p < 0.05; ** p < 0.01.
